# Lactic Acidosis in a Non-smoking Asthmatic Female: A Case of Salbutamol-Induced Elevated Lactate in the Context of Suspected Lower Respiratory Tract Infection

**DOI:** 10.7759/cureus.93796

**Published:** 2025-10-03

**Authors:** Tarunjog Singh Kalra, Zoha Naeem, Hania Afsar, Ragunath Durairajan

**Affiliations:** 1 Internal Medicine, Russells Hall Hospital, Dudley, GBR

**Keywords:** acute asthma management, asthma exacerbation, beta-2 agonist, high lactate in asthma, inhaled bronchodilators, non-hypoxic lactic acidosis, salbutamol-induced lactic acidosis, salbutamol toxicity, side effects, type b lactic acidosis

## Abstract

Lactic acidosis is typically associated with hypoperfusion states such as sepsis or shock; however, non-hypoxic mechanisms like beta-2 agonist therapy can also lead to lactic acidosis. We report the case of a 56-year-old female with asthma exacerbation who developed type B lactic acidosis following high-dose salbutamol administration. Clinical improvement and rapid normalisation of lactate after reducing bronchodilator use support the diagnosis of salbutamol-induced lactic acidosis (SILA). This report emphasises the importance of distinguishing SILA from more serious causes of hyperlactatemia, such as sepsis or pulmonary embolism.

## Introduction

Lactic acidosis is a critical biochemical derangement characterised by elevated serum lactate levels (>4 mmol/L) and acidemia (pH <7.35) [1]. It is broadly classified into type A, caused by tissue hypoxia (e.g., sepsis, hypovolemia, cardiac failure), and type B, which occurs without overt hypoperfusion and is often drug- or toxin-induced [2]. Excessive beta-2 agonist use has been associated with type B lactic acidosis due to increased glycolysis, lipolysis, and subsequent pyruvate accumulation [3,4]. 

Salbutamol, a short-acting beta-2 adrenergic receptor agonist, is widely used in the treatment of acute asthma exacerbations. However, high doses or continuous administration can cause significant metabolic disturbances, including hypokalemia, hyperglycaemia, and lactic acidosis [3,4]. The underlying mechanism is believed to involve enhanced intracellular cyclic AMP production, stimulating glycolytic flux and mitochondrial pyruvate overload [3].

Although rarely reported, salbutamol-induced lactic acidosis (SILA) may lead to misdiagnosis as sepsis or pulmonary embolism, potentially resulting in unnecessary investigations and treatment escalation [4]. We report a case of a middle-aged female who developed lactic acidosis following high-dose salbutamol administration for asthma, which resolved rapidly after the de-escalation of bronchodilator therapy.

## Case presentation

A 56-year-old female with a known history of asthma presented to the emergency department with worsening dyspnea, productive cough with green sputum, and generalised weakness for four days. She denied any chest pain, hemoptysis, or recent travel. Her medical history included hypothyroidism, anemia, Barrett’s esophagus, schizoaffective disorder, arthritis, vitiligo, diverticulosis, and a prior pulmonary embolism for which she was on rivaroxaban. She had self-initiated two consecutive courses of steroid-doxycycline therapy in the preceding fortnight without improvement.

**Figure 1:**
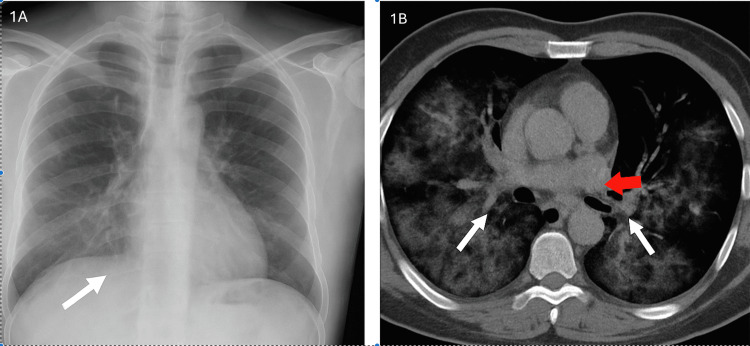
Imaging findings A. Chest X-ray demonstrates left mid-lower zone airspace shadowing (white arrow) consistent with possible consolidation. B. CTPA image reveals patchy bilateral ground-glass opacities (white arrows) and enlarged right hilar lymph node (red arrow). No evidence of pulmonary embolism is seen CTPA: computed tomography pulmonary angiogram

On examination, she was alert but in respiratory distress. Her respiratory rate was 20 breaths/minute, heart rate was 129 beats/minute, blood pressure was 139/98 mmHg, and oxygen saturation was 97% on 1 L/min of nasal oxygen. Chest auscultation revealed widespread expiratory wheeze. 

Initial venous blood gas revealed a pH of 7.37 (7.35-7.45), pCO₂ of 4.36 kPa (4.7-6.0), bicarbonate of 18.8 mmol/L (22-26), and lactate of 5.4 mmol/L (0.5-2.2). The elevated lactate on the initial venous blood gas was indeed obtained immediately upon presentation to the emergency department, before additional hospital-administered salbutamol therapy was given. The patient had already been using repeated doses of inhaled/nebulised salbutamol at home, which had likely contributed to the raised lactate before arrival.

After reducing the frequency of nebulised salbutamol in the hospital, lactate levels normalised within 24 hours. We have clarified this sequence of events in the Case Presentation section to make the timeline more explicit. Laboratory investigations showed a white cell count (WBC) of 11.2 ×10⁹/L and a C-reactive protein (CRP) of 17 mg/L. Renal and liver function tests were normal (Table 1). A chest X-ray (Figure 1A) demonstrated airspace shadowing in the left mid-lower lung zone. CT pulmonary angiography (CTPA) (Figure 1B) excluded pulmonary embolism but revealed patchy consolidation, ground-glass opacities, and right hilar lymphadenopathy. 

**Table 1:** Laboratory results WBC: white blood cells; CRP: C-reactive protein; ALT:: alanine aminotransferase; ALP: alkaline phosphatase

Test	Value	Reference range	Unit
WBC	11.2	4.0 - 11.0	×10⁹/L
CRP	17	<5	mg/L
Lactate (initial)	5.4	0.5 - 2.2	mmol/L
Lactate (day 2)	1.0	0.5 - 2.2	mmol/L
Creatinine	63	44 - 80	µmol/L
ALT	23	7 - 56	IU/L
ALP	88	40 - 129	IU/L

The patient received repeated back-to-back nebulisations of salbutamol and ipratropium, intravenous magnesium sulfate, oral prednisolone (40 mg daily), and levofloxacin (500 mg twice daily, due to penicillin allergy). Intravenous fluids (2 litres) were administered over 12 hours. Given her tachycardia and elevated lactate, sepsis or recurrent pulmonary embolism was initially suspected. However, her hemodynamic stability, mild inflammatory markers, and negative imaging ruled out these diagnoses. The patient received repeated back-to-back nebulisations of 2.5 mg salbutamol with 500 µg ipratropium every 20-30 minutes over the first two to three hours in the emergency department, in addition to multiple doses of salbutamol inhaler at home before arrival.

By day two, her symptoms improved significantly. Nebulisation therapy was discontinued. Repeat arterial blood gas showed normalisation of lactate (1.0 mmol/L), pH of 7.46, pCO₂ of 4.17 kPa, and bicarbonate of 22 mmol/L. She was discharged home in a stable condition.

## Discussion

The presence of lactic acidosis in asthma exacerbations often raises concern for sepsis or thromboembolism. In our patient, these possibilities were appropriately considered but excluded based on stable hemodynamics, low inflammatory markers, and negative imaging results. Her subsequent improvement after reducing the frequency of salbutamol strongly supported a diagnosis of SILA. Beta-agonists such as salbutamol stimulate β₂-receptors, leading to increased intracellular cyclic AMP, activation of glycolytic enzymes, and enhanced conversion of glucose to pyruvate and lactate [3]. Excessive use can overwhelm mitochondrial oxidative capacity, resulting in lactate accumulation even in well-oxygenated tissues [3]. Literature indicates that both racemic salbutamol and levosalbutamol (the R-enantiomer) can potentially induce lactic acidosis, though some studies suggest levosalbutamol may have a lower incidence of metabolic side effects. However, cases of levosalbutamol-induced lactic acidosis have also been reported.

Miranda et al. reported similar cases of lactic acidosis in status asthmaticus, identifying beta-agonist overuse as a frequent precipitant [3]. Najout et al. described severe SILA in a patient receiving high-dose salbutamol, with rapid resolution after de-escalation [4]. Khanal et al. highlighted non-hypoxic hyperlactatemia due to the Warburg effect in a lymphoma patient, underlining the importance of distinguishing type B causes [5].

When compared with these reports, our case demonstrates several consistent features: normal oxygenation, absence of sepsis, and resolution of hyperlactatemia upon tapering salbutamol. However, unlike Najout et al.’s case, which required intensive care monitoring, our patient responded to a relatively modest adjustment in inhaled therapy. This suggests that SILA may present across a spectrum of severity, influenced by the cumulative beta-agonist dose, route of administration, and individual metabolic variability. Based on prior reports, “high-dose” β-agonist therapy is variably defined, but commonly refers to continuous nebulisation or frequent intermittent doses exceeding 10-15 mg of salbutamol within 24 hours [4,6]. Our patient’s regimen, which included multiple consecutive nebulisations totalling approximately 7.5-10 mg over a short period, falls within the range associated with reported cases of β-agonist-induced lactic acidosis.

In a systematic review by Meert et al., beta-agonist therapy was identified as a significant contributor to lactic acidosis in both pediatric and adult status asthmaticus, with prevalence underestimated due to frequent misattribution to sepsis [6]. Similarly, Colombier et al. demonstrated that even standard nebulised doses of salbutamol can transiently increase lactate levels in children, although usually without clinical consequence [7]. These findings highlight the importance of clinical context - lactate elevation in asthma should not automatically trigger aggressive sepsis protocols.

Our case, therefore, aligns with prior literature in reinforcing the concept that SILA is a diagnosis of exclusion but a crucial one to consider. Early recognition not only prevents unnecessary ICU admission, broad-spectrum antibiotic use, and repeated imaging but also supports more judicious titration of bronchodilator therapy. In cases of suspected SILA, current evidence suggests that β-agonists should not be abruptly discontinued, as they remain essential for bronchodilation in acute asthma. Instead, de-escalation to the lowest effective dose is recommended while optimising adjunctive therapies such as systemic corticosteroids, intravenous magnesium sulfate, and anticholinergics [4,6].

## Conclusions

This report highlights the importance of recognising lactic acidosis as a potential side effect of excessive beta-agonist use in asthma exacerbations. Prompt identification and supportive management can lead to favourable outcomes. Clinicians should consider SILA in cases of unexplained hyperlactatemia in otherwise stable asthma patients. Awareness of this condition is crucial, as it can prevent misdiagnosis and unnecessary interventions. Early recognition may allow clinicians to adjust therapy appropriately and avoid unnecessary investigations. A better understanding of the mechanisms behind SILA can support safer use of beta-agonists in acute asthma, although further studies are needed to confirm its impact on outcomes.
